# Dry Season Melioidosis in the Tropical North of Australia

**DOI:** 10.3390/pathogens15070726

**Published:** 2026-07-09

**Authors:** Marisia Madrigal-Solis, Mirjam Kaestli, Mark Mayo, Celeste Woerle, Ella M. Meumann, Bart J. Currie

**Affiliations:** 1 Menzies School of Health Research, Charles Darwin University, Darwin, NT 0810, Australia; 2 Territory Pathology, Royal Darwin Hospital, Darwin, NT 0810, Australia; 3 Department of Infectious Diseases, Royal Darwin Hospital, Darwin, NT 0810, Australia

**Keywords:** melioidosis, *Burkholderia pseudomallei*, climate, dry season, rainfall, environmental exposure, anthropogenic environmental disturbance, climate change

## Abstract

**Background:** Melioidosis correlates strongly with rainfall, and there is substantial diversity in climate between melioidosis-endemic locations. The Northern Territory of Australia epitomises the “wet/dry” tropics, with a prolonged dry season from May to October. We analysed dry season cases of melioidosis during 35 consecutive years and compared these with wet season cases. We aimed to provide insights into how dry season cases of melioidosis may occur in this region and explore non-rainfall exposures that are usually not considered in the wet season. **Methods:** Case epidemiological and clinical data were extracted from the Darwin Prospective Melioidosis Study. Weather parameters, including daily rainfall, were analysed using generalised additive models and conditional logistic regressions to assess associations between dry season cases and preceding rainfall. **Results:** Of 1520 melioidosis cases between 1989 and 2024, there were 325 (21%) in the dry season. While the well-recognised clinical diversity of melioidosis was also seen amongst dry season cases, pneumonia was proportionally less common and cutaneous melioidosis was more common than in the wet season. A total of 23% of dry season patients had no identified clinical risk factors for melioidosis, compared to 14% in the wet season. Mortality was 8% in the dry season and 11% in the wet season. There was a range of plausible explanations for many of the dry season cases, including unseasonal rainfall prior to infection. Infections in urban settings were notable, with anthropogenic factors such as irrigation and construction resulting in persistence of *Burkholderia pseudomallei* in the environment during the dry season. A total of 3% of cases remained unexplained. **Conclusions:** Not all dry season cases are explained by infection occurring the previous wet season or by unseasonal rainfall in the dry. Identification of cases in the dry season support the need for year-round prevention strategies during potential exposure to contaminated water or soil. Further prospective studies are needed to better define the infecting events resulting in melioidosis, especially in the dry season. These studies should include timely history taking from the case and their family and selected environmental sampling for *B. pseudomallei*.

## 1. Introduction

Melioidosis is the disease following infection with *Burkholderia pseudomallei.* Originally described in Southeast Asia and northern Australia, melioidosis is increasingly being unmasked in other tropical and subtropical locations. It has been documented to be expanding to new locations, most recently in the southern continental United States [1,2]. *B. pseudomallei* is notable as a saprophytic soil-dwelling gram-negative free-living bacterium with a heterogeneous distribution in soil and water in tropical and subtropical climates [3,4,5]. As a sapronotic pathogen, it causes disease in both humans and animals, but zoonotic transmission from animals to humans is extremely uncommon [4].

Nosocomial and laboratory-acquired melioidosis, vertical transmission in utero, person-to-person transmission, such as through breast milk in mothers and animals (especially goats) with melioidosis mastitis and infection from products contaminated with *B. pseudomallei* are all described but exceptionally rare [3,4,6]. Each case of melioidosis in endemic locations is a point source infection of a human or animal from exposure to *B. pseudomallei* in the local soil or water. The route of infection can be percutaneous, inhalation of *B. pseudomallei* in aerosols generated during weather events or ingestion, such as with untreated water supplies contaminated with *B. pseudomallei* [7,8,9,10].

The epidemiology of melioidosis in endemic regions is driven largely by weather factors, most importantly annual rainfall patterns and occurrence of severe weather events such as hurricanes, cyclones and typhoons. While in Singapore, rainfall and cases of melioidosis occur throughout the year [11], northeast Thailand and the Northern Territory of Australia are notable for distinctive wet and dry seasons, where in both regions about 20% of cases of melioidosis occur in the dry season [12,13,14]. While the wet season predominance of cases of melioidosis reflects in large part that *B. pseudomallei* proliferates in soil and surface water once the rains begin [10], there is a natural persistence of *B. pseudomallei* through the dry season in deeper soil layers. Prolonged dry periods seem to favour the *B. pseudomallei* staying in deeper soil, and initial heavy rainfall at the start of the wet season helps more towards surface distribution and later aerosolisation of the bacteria [11,15,16]. This persistence can also be anthropogenically influenced, such as with the irrigation of surface soils [17], and with untreated water systems, such as bores (water wells) with holding tanks [18].

In this study, we have investigated the epidemiology, clinical parameters and exposure factors of all dry season cases of melioidosis from the tropical Northern Territory over 35 years, with the aim of better understanding how, why and when infection with *B. pseudomallei* occurs for individuals presenting during the dry season. This is the first study to focus solely on melioidosis cases in the dry season and aims to contribute towards climate-driven and anthropogenic influences on cases of melioidosis.

## 2. Materials and Methods

This was a retrospective cohort study of data from the Darwin Prospective Melioidosis Study (DPMS) from the tropical Top End of the Northern Territory (NT) of Australia, a region with high incidence rates of melioidosis [14]. Darwin epitomises the distinctive “wet-dry” tropics: median rainfall in the wet season (1st of November to 31st of April) in Darwin each year between 1990 and 2024 was 1682.6 mm^3^, compared with 83.6 mm^3^ in the dry season (1st of May to 31st of October). The DPMS has collected data on NT melioidosis cases since 1989 and is an ongoing study. It encompasses clinical and laboratory data for all patients with a culture-confirmed diagnosis of melioidosis [12], with the most recent publication reporting 30 years of data up to 2019 [14]; this study of dry season melioidosis covers 35 years of data up to 2024. In DPMS data collection, specific data on potential exposure events are freehand text added to the DPMS patient data sheet, taken from the patient history obtained during admission and clinical follow-up. For this dry season study, the original prospectively recorded data on potential specific events were augmented retrospectively by further analysis of patient records for dry season cases. In the DPMS, acute melioidosis is defined as symptoms for less than two months prior to diagnosis, chronic melioidosis as symptoms for more than two months, and activation from latency as new culture-confirmed clinical disease with evidence of prior asymptomatic infection with *B. pseudomallei*.

Dry season cases were defined as cases diagnosed between 1st of May and 31st of October, and wet season cases were diagnosed between 1st of November and 30th of April. The date of diagnosis was the date that *B. pseudomallei* was first identified from a patient’s clinical sample (blood, sputum, urine, skin swab, pus, cerebrospinal fluid). Patient demographics, clinical risk factors for melioidosis (comorbidities), potential recreational and/or occupational exposure, potential specific infection events and clinical presentations and outcomes were all defined as previously described for DPMS [14]. Comparisons between the wet and dry seasons were made and summarised using a chi-square statistic [19].

To explore the association between dry season cases and incidence, population data were retrieved from the Australian Bureau of Statistics (ABS) for the yearly estimated resident population for the capital city of Darwin in the Northern Territory [20]. Information on monthly rainfall to correlate with all dry season cases in the Top End was retrieved from the Australian Bureau of Meteorology (BOM) website, https://www.bom.gov.au/climate/data/index.shtml (accessed on 10 September 2025). To explore associations between dry season cases in urban and rural Darwin and preceding rainfall, daily rainfall data (mm) and average humidity at 15:00 hours were obtained from the Australian Bureau of Meteorology (BOM) for the Darwin Airport weather station (station number 014015) [21]. For these analyses, days with acute dry season cases from the urban and rural Darwin region (N = 93) between May 20th and 31st October were included; early May cases between May 1st and May 20th, inclusive, were excluded because these infections could plausibly reflect exposure during the end of the preceding wet season. We previously defined the incubation period, that is, the time from a suspected infecting event to symptom onset in the DMPS to be 1 to 21 days, median 4 days [4,14]. Cases with travel history that may have reflected the infection event were also excluded (N = 8). Where multiple cases occurred on the same date (N = 2), these were treated as a single case-day. Conditional logistic regression was used to assess whether case-days were more likely than matched control-days to have experienced a rainfall event (>0.1mm) in the two or four weeks preceding diagnosis. The choice of rainfall in the two weeks preceding diagnosis was to cover the noted incubation period, and the choice of 4 weeks was to cover both incubation period and the possibility of a somewhat delayed presentation after onset of symptoms. For each case-day, five control dates were randomly selected from the same dry season, with a minimum separation of 7 days between sampled control dates to reduce overlap in the antecedent cumulative rainfall windows of the control dates. Analyses were performed in R Statistical software (Version 4.5.3 from 2026) using the survival package [22,23].

In addition, binomial generalised additive models (GAMs) were fitted to further assess temporal patterns in dry season case-days. Predictors included smooth terms for day into the dry season and year, together with cumulative rainfall in the preceding two or four weeks. Models were fitted using restricted maximum likelihood (REML) with the mgcv package [24]. Model diagnostics were assessed using the DHARMa package [25], including checks for lack of residual patterns against fitted values and predictors and no temporal correlations. Negative binomial GAMs with a Darwin population offset (log transformed) were used to estimate annual dry-season incidence rates. The influence, if any, of rainfall during the preceding wet season (1st of November to 30th April) was also assessed.

This study was approved by the Human Research Ethics Committee of the Northern Territory Department of Health and the Menzies School of Health Research (HREC 02/38), found in the Supplementary Materials.

## 3. Results

### 3.1. Darwin Prospective Melioidosis Study (DPMS)

Between 30 November 1989 and 31 October 2024, 1520 episodes of melioidosis were recorded from 1439 individuals in the DPMS. Cases included both first episodes of melioidosis and cases of recurrent melioidosis, as previously defined [14]. First admission cases were predominantly acute cases, N = 1280 (89%), followed by chronic cases defined as symptoms present for two or more months, N = 129 (9%), and activation from latency N = 29 (2%) [26]. Recurrent melioidosis in individuals with prior melioidosis, as assessed by genotyping of *B. pseudomallei* [14], included relapsed cases, N = 55 (67%), and new infections, N = 27 (33%).

### 3.2. Dry Season Cases

Out of 1520 DPMS cases, 325 (21.4%) were admissions during the dry season. Table 1 summarises demographic data and the nature of melioidosis in the wet and dry seasons. Of dry season patients, 62% were male, and 5% were children under 15 years. The mean age of presentation was 49 years, with a range between 9 months and 91 years. First Nations Australians accounted for 47% of dry season cases in comparison to 55% of wet season cases (*p* = 0.013). First admissions in the dry season included acute cases, N = 203 (73%), 58 chronic cases, N = 58 (20%), and activation from latency cases, N = 21 (7%). One case was excluded from categorisation of first admission because he had an initial episode of melioidosis prior to the commencement of the DPMS. Second, and in one instance a third admission, consisted of 39 cases of relapsed melioidosis and 4 individuals who had new infections as confirmed by genotyping of *B. pseudomallei*.

While acute melioidosis cases accounted for 73% of cases in the dry season, this was significantly lower than the 93% wet season acute cases (*p* < 0.01) (Table 1). This contrasts with chronic melioidosis, activation from latency and relapsed presentations all being significantly more common in the dry season (*p* < 0.01) (Table 1).

Table 2 shows a summary of comorbidities, exposure history, primary clinical presentations, and outcomes for those presenting with acute melioidosis, comparing dry and wet seasons. Of the 203 dry season cases with acute melioidosis, N = 156 (77%) had at least one clinical risk factor (comorbidity), consistent with DPMS overall data. Although diabetes was still present in N = 83 (41%) of dry season acute melioidosis cases, a significantly greater proportion had no identified comorbidity (N = 46 (23%) compared with N = 151 (14%) for wet season acute melioidosis). While pneumonia was the most common clinical presentation in both seasons, it was substantially more common in the wet season (N = 627 (58%) compared with N = 61 (30%) for dry season acute melioidosis, *p* < 0.01). Conversely, cutaneous melioidosis, septic arthritis, and osteomyelitis were proportionally significantly more common presentations in the dry season (Table 2). Dry season acute melioidosis cases were often still unwell on presentation, bacteraemia was present in N = 99 (49%), septic shock in N = 38 (19%), intensive care management was required for N = 50 (25%) and ventilation for N = 27 (13%). Overall, of the total 203 dry season acute cases, N = 16 (8%) died from their melioidosis, in comparison to N = 122 (11%) of the 1077 wet season cases with acute melioidosis (*p* = 0.01).

Travel outside of the Northern Territory as a potential exposure event for inoculation and infection of *B pseudomallei* was documented in 8 dry season cases, of which 7 travelled to Southeast Asia, where the wet season coincides with the NT dry season. In one case, infection was considered likely to have occurred in northern Western Australia.

### 3.3. Environmental Exposure Events During the Dry Season

Recreational exposure and occupational exposure were documented for N = 172 (85%) and N = 31 (15%) of dry season acute cases, respectively, similar to wet season cases (Table 2). On review of the case histories, there were N = 46 (23%) dry season cases with acute melioidosis where a specific potential infecting event was noted and described, with exposure to soil and/or water. Examples where there was considered a likelihood of a specific inoculating event included: exposure to contaminated bore water, such as drinking, showering and washing of skin wounds; mud and wet soil exposure was wide-ranging, including activities in irrigated sports fields, walking barefoot and playing in irrigated domestic yards, parks, and along creek lines, sometimes with documented skin trauma; both recreational and occupational garden, yard, and agricultural exposures were common, such as mowing lawns, use of whipper snippers (weed whacker), potting plants, digging holes and irrigation maintenance, high-pressure hosing (power-washing), and in one case collecting soil samples without personal protective equipment; several dry season cases described being caught outside in unseasonal brief but heavy rainfall; and one notable case involved a motor vehicle accident with a lower limb fracture and multiple asphalt abrasions, with subsequent melioidosis after admission to hospital.

### 3.4. Darwin Cases of Melioidosis and Weather Correlation

**Figure 1:**
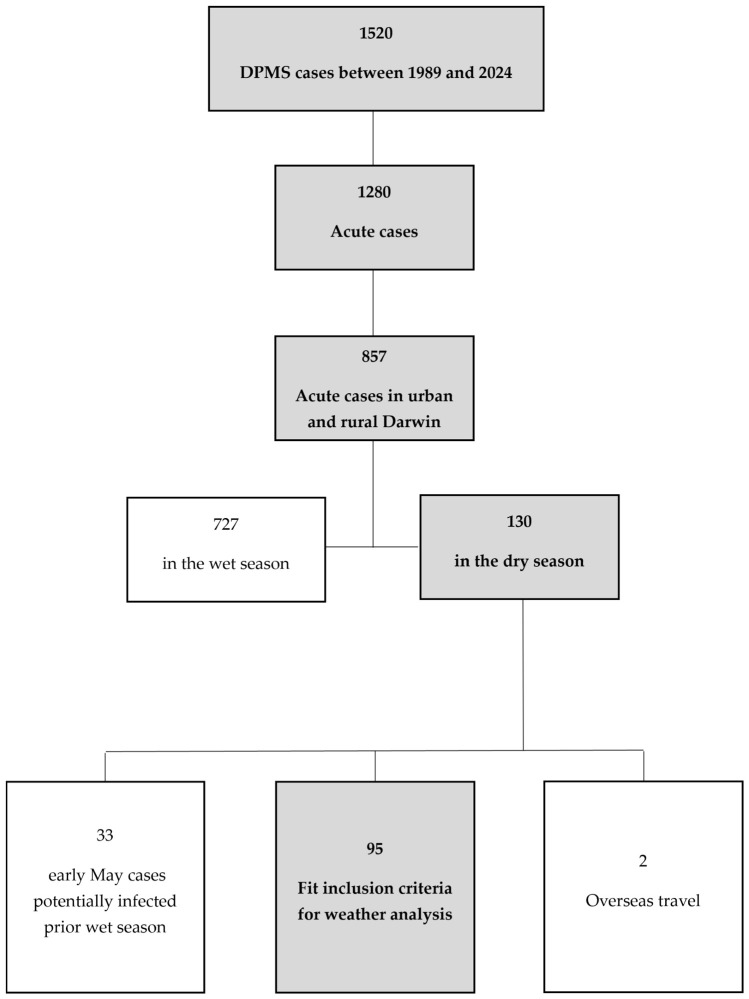
Distribution of cases from the DPMS for the weather analysis focusing on acute dry season cases in urban and rural Darwin.

**Figure 2:**
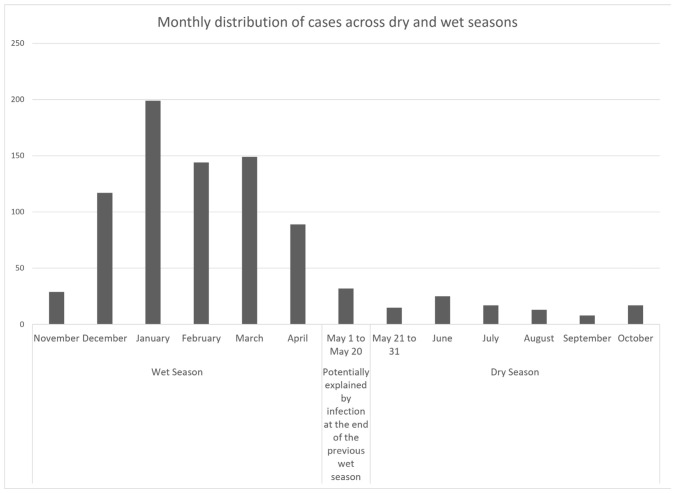
Monthly distribution of acute melioidosis cases in urban and rural Darwin (1989–2024).

**Figure 3:**
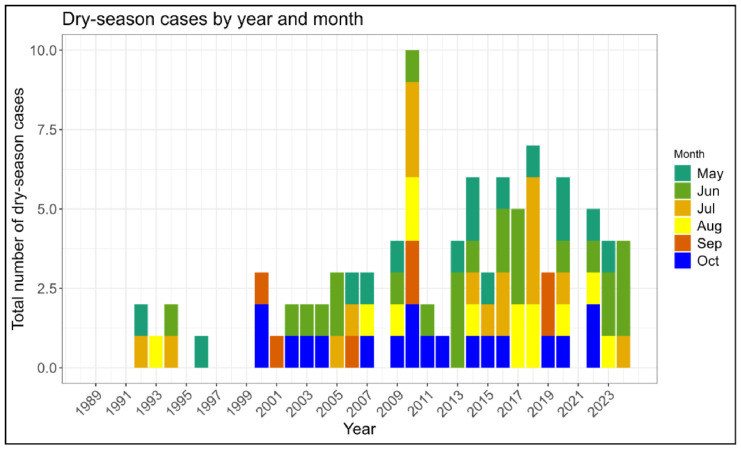
Yearly and monthly distribution of acute melioidosis cases in urban and rural Darwin in the dry season (cases between May 1st and May 20th were excluded from weather analysis).

**Figure 4:**
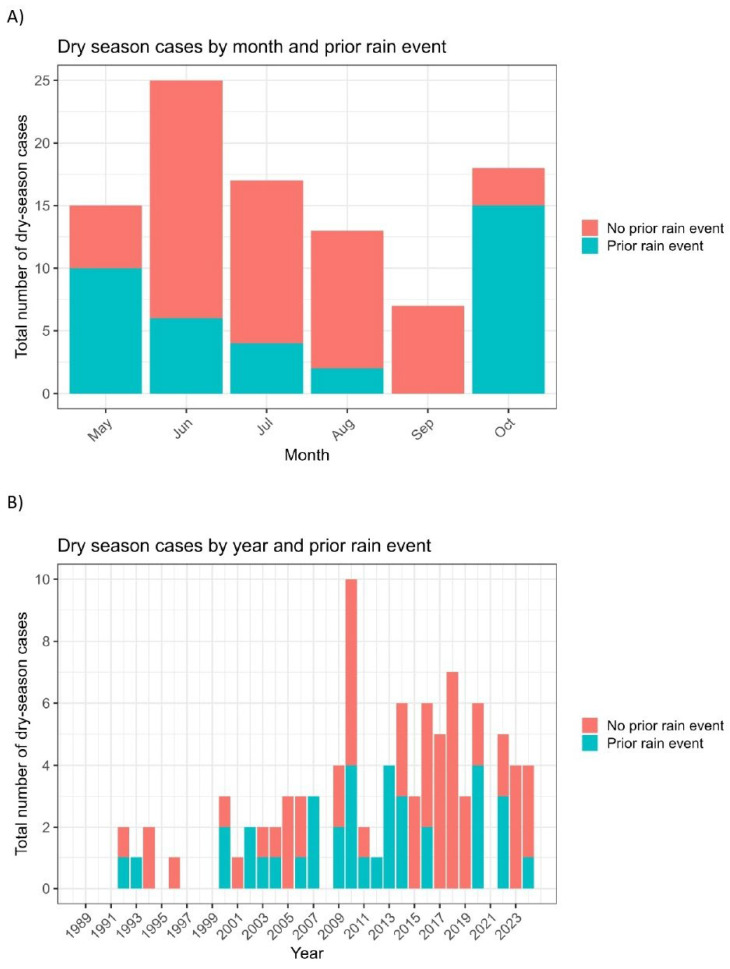
Exposure to rain in (**A**) the previous two weeks (rain > 0.1 mm) before diagnosis by month and (**B**) in the previous two weeks (rain > 0.1 mm) by year for acute melioidosis cases in the dry season in the urban and rural Darwin areas (cases between May 1st and May 20th were excluded from weather analysis).

For the cases with acute melioidosis, 130 resided in the urban and rural Darwin region; see Figure 1. Figure 2 shows the monthly distribution of these cases over the 35 years of the study. A total of 33 cases diagnosed in May, up to May 20th inclusive, were excluded from the weather analysis as noted in Methods, being potentially attributable to exposure late in the preceding wet season. A further two cases were excluded as they had a history of overseas travel. Two dates had two reported cases each date. Therefore, 93 case-days were included in the weather correlation analysis for 95 cases (Figure 1), and Figure 3 shows the yearly and monthly distribution of these. Conditional logistic regression findings were consistent across the 2-week and 4-week rainfall exposure windows, with neither showing a significant association between antecedent rainfall and dry-season case-days (*p* > 0.1 for both). Neither did average humidity at 15:00 hours show an effect (*p* > 0.1). Figure 4 summarises exposure to rain (>0.1 mm) in the two weeks before diagnosis, shown by month (4A) and by year (4B). Although conditional logistic regression did not show a significant association between prior rainfall and dry-season case-days, Figure 4A indicates that rainfall-associated cases were more common at the beginning of the dry season and again towards the end in October, coinciding with the first wet-season storms.

**Figure 5:**
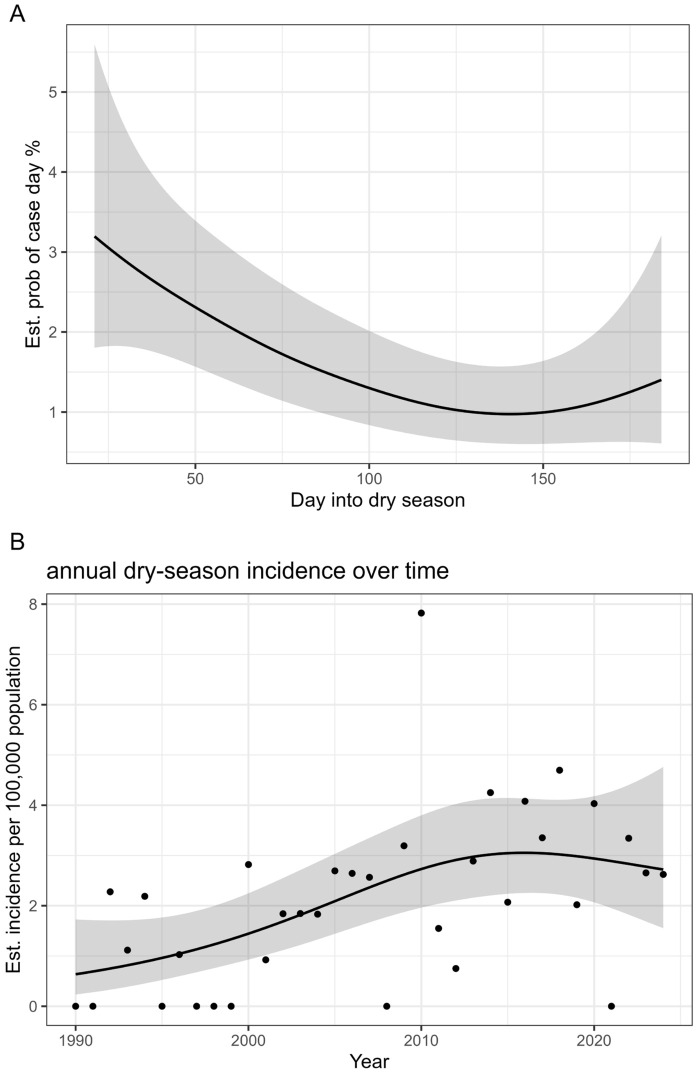
Estimated smooth effects from GAMs showing (**A**) the probability of a dry season case-day across the dry season (binomial GAM) and (**B**) annual dry season melioidosis incidence rates over time (negative binomial GAM). Grey shading indicates 95% confidence intervals. Rainfall was not associated with the outcome in either model: neither cumulative rainfall in the preceding four weeks in panel A nor total rainfall in the preceding wet season in panel B. Full model results are provided in Supplementary Table S1.

Generalised additive models (GAMs) estimated a significant decline in the probability of a dry season case-day as the dry season progressed (Figure 5A), while annual dry season incidence rates increased through 1990 to 2010 and then stabilised in the following decade (Figure 5B). Total rainfall in the preceding wet season was not associated with annual dry season incidence rates.

### 3.5. Explanations for Dry Season Melioidosis

**Figure 6:**
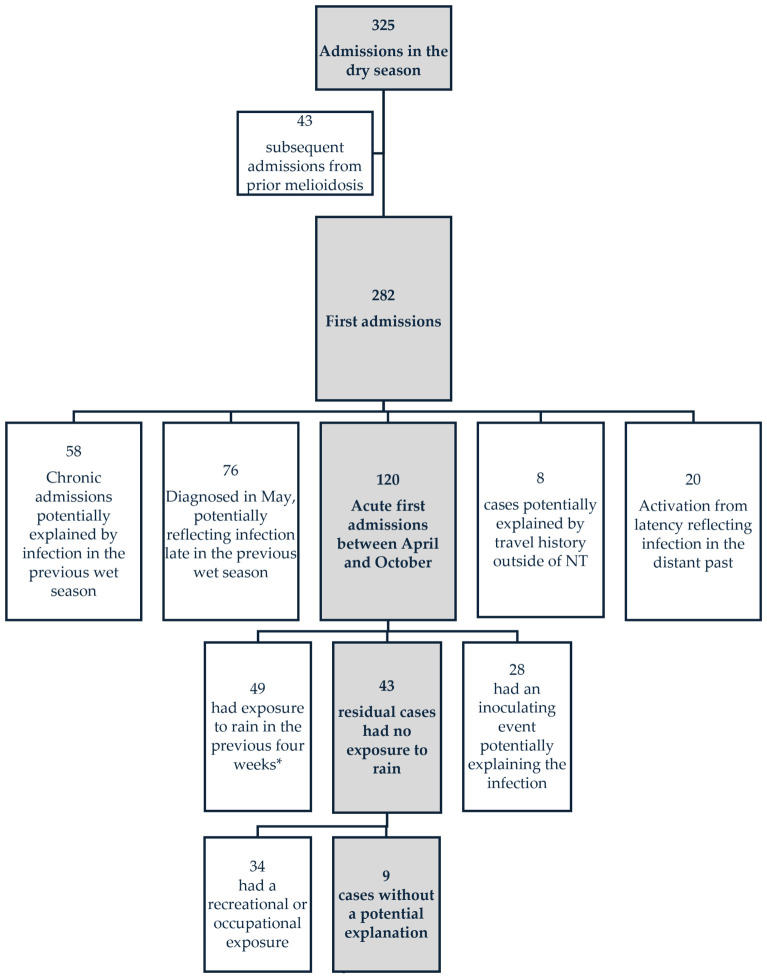
Proposed potential explanations of cases in the dry season. From the initial 325, firstly, subsequent admissions were removed, leaving 282 first admissions. First admissions were filtered into cases with known travel history, chronic cases, activation from latency cases, and acute in May in order to focus on acute cases between April and October, the rest of the dry season. Acute dry season cases were then further categorised into an inoculating event, exposure to rain or no exposure. Of those with no exposure, we compared against recreational and occupational exposure before arriving at those with no explanation. * Rainfall exposure analysis for the residual unexplained cases showed 49 with exposure to rainfall and 43 with no exposure to rainfall.

Figure 6 shows proposed potential explanations for the 325 dry season cases of melioidosis in the DPMS. Explanations included: recurrent melioidosis from a prior admission (N = 43); chronic melioidosis (N = 58), usually representing a late presentation of infection during the prior dry season; activation from latency from prior infection (N = 21); a described potential specific infection event (N = 28); and admission during all of May, potentially reflecting infection late in the prior wet season (N = 77). Of the residual 47, 37 had a history of recreational and/or occupational exposure. This left 9 cases (3%) without a potential explanation for their dry season melioidosis presentation.

## 4. Discussion

### 4.1. Demographics and Clinical Parameters

Recent articles on melioidosis have confirmed the influence of a combination of environmental exposure, host susceptibility, and climatic variability [3,4,27]. All three of these factors impact the occurrence of melioidosis during the 6 months of the dry season in the tropical Northern Territory, which accounts for 21.4% of all cases despite the very distinctive dry 6 months seen in the “wet/dry” tropics. Acute melioidosis remains the most common nature of presentation (72%) in the dry season, but chronic melioidosis and activation from latency are both significantly more common in the dry season than in the wet season (Table 1).

Chronic cases have been sick for 2 months or longer (median 4 months and range 3–36 months) [28], and therefore can often reflect infection during the prior wet season, and activation from latency can be expected not to be seasonally dependent [26]. The DPMS cases attributed to activation from latency were more common in the dry than the wet season (Table 1), which likely reflects an under-ascertainment in the wet season, when cases are unsurprisingly attributed to the frequent wet season rains. This reflects the difficulty of getting an accurate timeframe of activation from latency of melioidosis in endemic regions, as previously discussed [26]. In addition, relapsed melioidosis reflects failure of eradication of *B. pseudomallei* and can also be expected not to be seasonally driven [28]. Nevertheless, there were more cases of relapsed melioidosis in the dry season, and this likely reflects that relapses are within 1–12 months after completion of therapy and therefore often occur in the dry season following initial melioidosis diagnosis in the prior wet season.

Patients with acute melioidosis may have had symptoms for up to two months prior to diagnosis. Many diagnosed cases in May were likely infected by *B. pseudomallei* in the prior wet season, hence the exclusion of cases up to May 20th from the weather analysis.

While the most common presentation of melioidosis in the dry season remained pneumonia (30%), it was half as common as wet season presentations (58%) (Table 2). This, at least in part, reflects the influence of severe weather events (only in the wet season), resulting in aerosolised *B. pseudomallei* implicated in inhalation and subsequent pneumonia [11,14,15,29]. Conversely, cutaneous melioidosis was significantly more common in the dry season (22% of cases) (Table 2), likely reflecting two aspects of the epidemiology: firstly that, although no individual risk factor (comorbidity), including diabetes, was significantly more common between the seasons, people with no identified risk factor were significantly more common in the dry season (23%); secondly that inhalation of aerosolised *B. pseudomallei* would be extremely unlikely in the dry season, where percutaneous infection is likely the dominant mode of infection. Percutaneous infection can still result in bacteraemia, sepsis and death, but healthy individuals without classical risk factors for melioidosis often control the infection at the site of skin inoculation without bacterial spread and systemic illness [30,31]. Therefore, cutaneous melioidosis usually follows a more indolent course, reflected in it being a common presentation of chronic melioidosis [28], and that cases of cutaneous melioidosis from the wet season can present in the subsequent dry season.

Of note was that, while there was a trend for less bacteraemia in dry season cases (Table 2), intensive care management was not less commonly needed (25%). Overall, dry season mortality for those with acute melioidosis was 8% in comparison to 11% for the wet season acute cases (*p* = 0.01).

### 4.2. Seasonality and Rainfall

In Taiwan, Mu and colleagues identified 322 cases between 2000 and 2011, reporting that cases in the wet season occurred five times more frequently than in the dry season [32], consistent with our past [14,33] and current findings from tropical northern Australia. Irrespective of the variety of seasonal patterns across regions endemic for melioidosis, the universal finding is a strong association of cases of melioidosis with rainfall, with increased cases both at the start of the wet season and after extreme weather events [11,14,33,34]. For instance, in Singapore, where seasonality is less starkly defined than in the “wet-dry tropics”, the correlation of cases of melioidosis with rainfall and severe weather events is still strong [11,35].

The link between rainfall and melioidosis cases is considered to reflect a greater abundance of *B. pseudomallei* in shallow soil layers and surface water after rain. This reflects the redistribution of the bacteria from deeper soil with a rising water table [36], and likely also increased bacterial persistence and replication under favourable post-rain conditions, including in the rhizosphere of actively growing grasses [37]. More than 90% of herbaceous biomass in the wet-dry tropical savannas is produced during the wet season [38,39]. In addition, aerosolisation of *B. pseudomallei* is found during severe weather events, with the recognised concern of this resulting in potentially fatal inhalational melioidosis [15,16,29,33,34,40]. Modelling suggests that climate change is likely to make climatic and environmental conditions more conducive to melioidosis, with potential future spread to locations outside the current endemic regions [33,41,42].

Anthropogenic factors also need consideration with respect to the burden of *B. pseudomallei.* For example, Hong Kong [43] and Singapore [11,35] are dense urban populations with gardens, building sites, sporting ovals and some limited rural locations, where potential anthropogenic environmental factors such as irrigation and construction have parallels with what is seen in Darwin [7,18,44] and in Cairns in far north Queensland [45] in Australia. These factors likely account for some dry season cases in our study, where there is exposure to and infection with *B. pseudomallei* present in irrigated soils and construction sites.

In our study, we noted that there were proportionally fewer First Nations Australians and cases from outside the Darwin region during the dry season (Table 1). This may well reflect that the anthropogenic influences on the presence and persistence of *B. pseudomallei* in Darwin during the dry season [44] are not seen in the rural and remote communities of the Northern Territory, where traditional lifestyles are maintained.

### 4.3. Environmental Exposures and Infecting Events

While exposure to wet season soils and surface water is the expected infecting event for most wet season cases of melioidosis, the reasons for dry season cases of melioidosis require additional considerations. As already noted, chronic melioidosis, activation from latency and recurrent melioidosis have cogent explanations for dry season presentation. In addition, even for acute melioidosis, cases early in the dry season may reflect exposure and infection late in the prior wet season, with up to 21 days being the reported incubation period for acute melioidosis and, according to our definition of ‘acute’, symptoms for up to two months prior to diagnosis [12]. Unseasonal rainfall during the dry season may also explain some cases. Specific activities were identified in some cases that provided a potential explanation for an infecting event during the dry season. These included gardening, lawn mowing and walking barefoot in heavily irrigated areas, mud exposure, through sporting ovals, digging, working on irrigation systems and power-washing. Skin injuries can also be a factor while swimming or fishing in creeks or during activities on sports fields. Additionally, exposure through ingestion of unchlorinated bore water was noted. Nevertheless, even with a liberal acceptance of such explanations, 3% of our dry season cases remained unexplained.

### 4.4. Limitations

There are important limitations in this study. Firstly, the analysis defined a fixed dry season from May 1st to October 31st for the 35 years of the study. While this is generally robust for the Top End of the Northern Territory, some year-to-year onset and offset variations of the rains do occur. Secondly, while the epidemiological and clinical data were collected prospectively, these were analysed retrospectively, and documented potential exposure events were not followed up for verification. Therefore, some of the proposed potential explanations for cases in the dry season must be considered speculative, and patient recall bias is also always possible. In addition, both information bias and diagnostic bias are possible limitations in a study spanning 35 years. Thirdly, identified potential environmental exposures were not routinely followed up with environmental sampling to identify and confirm the environmental presence of *B. pseudomallei* in the implicated location. Previous studies in small “hotspots” in Thailand, Vietnam and Australia have undertaken soil sampling to identify *B. pseudomallei* in the implicated environment and then used bacterial genotyping to confirm strain relatedness [8,9,46,47,48]. Finally, the rainfall analysis required assumptions about each patient’s likely time of infection and relied on data from a single weather station in Darwin.

### 4.5. Recommendations and Prevention Strategies

Confirmed cases of melioidosis in the dry season show that while the risk of melioidosis is substantially lower, prevention strategies should be considered throughout the year, and not only during the wet season. Tailored prevention strategies are recommended, including the use of treated water, closed footwear, gloves when gardening, and washing then covering open wounds. General infection prevention strategies should also be continuously practised. Improving awareness of the disease is an important part of prevention strategies [49,50,51,52,53].

## 5. Conclusions

In the wet/dry tropics of northern Australia, 20% of all first-episode melioidosis cases occurred during the six months of the dry season. While the well-recognised clinical diversity of melioidosis was also seen amongst dry season cases, pneumonia was proportionally less common and cutaneous melioidosis more common than in the wet season. A higher proportion of dry season patients (23%) had no identified clinical risk factors for melioidosis, and mortality in dry season melioidosis cases was 8%. There is a range of plausible explanations for many of the dry season cases, including infections in urban settings, where anthropogenic factors such as irrigation and construction result in persistence of *B. pseudomallei* in the environment. Nevertheless, at least 3% of cases in this study had no evident explanation for how they may have been infected, with unknown exposure pathways likely to exist. Further prospective studies are needed to better define the infecting events resulting in melioidosis, especially in the dry season. These studies should include timely history taking from the patient and their family, as well as selected environmental sampling for *B. pseudomallei*, with genotyping of clinical and environmental bacteria.

## Figures and Tables

**Table 1:** Demographics and nature of disease for 1520 consecutive cases of melioidosis.

	Dry Season	Wet Season	Total	*p*-Value
All Admissions	325	1195	1520	
First Admissions	283 (20% of all first admissions) *	1156 (80% of all first admissions)	1439	
Second Admissions	43	39		
Demographics **	N = 283	N = 1156	N = 1439	
Female	108 (38%)	441 (38%)	616	
Male	175 (62%)	715 (62%)	823	
Age Range ***	9 m–91 y	7 m–97 y	7 m–97 y	
First Nations	132 (47%)	634 (55%)	766	0.0132
Region				
Darwin urban and rural	136 (48%)	652 (56%)	788	
Darwin regional	50 (18%)	134 (12%)	184	
NT Remote	95 (34%)	358 (31%)	453	
Other	2 (1%)	12 (1%)	14	
First Admission Diagnosis *				
Acute	203 (73%)	1077 (93%)	1280	<0.01
Chronic	58 (20%)	71 (6%)	129	
Activation from latency	21(7%)	8 (<1%)	29	
Subsequent Admission Diagnosis	N = 43	N = 39		
Relapse	39 (91%)	16 (41%)	55	<0.01
New infection	4 (9%)	23 (59%)	27	

* One patient had their first episode of melioidosis before the DPMS study began, so data is unknown on diagnoses for that admission. ** Based on first admissions. *** Age at first admission with melioidosis.

**Table 2:** Differences in clinical risk factors (comorbidities), exposure history and presentations of acute melioidosis cases in the dry and wet seasons.

	Dry Season		Wet Season		*p*-Value *
Total Case, N = 1280	203	16%	1077	84%	
Risk Factors					
Diabetes	83	41%	523	49%	0.045
Hazardous Alcohol Use	79	39%	437	41%	0.658
Chronic Kidney Disease	17	8%	140	13%	0.065
Immunosuppression	18	9%	106	10%	0.667
No risk factors	46	23%	151	14%	0.002
Occupational exposure	31	15%	160	15%	0.880
Recreational exposure	172	85%	859	80%	0.101
Documented specific potential exposure event	46	23%	*		
Clinical Presentation					
Pneumonia	61	30%	627	58%	<0.001
Cutaneous melioidosis	44	22%	90	8%	<0.001
Genitourinary melioidosis	35	17%	132	12%	0.053
Bacteraemia with no evident focus	20	10%	122	11%	0.539
Septic arthritis	13	6%	20	2%	<0.001
Soft tissue infection	10	5%	38	4%	0.336
Neurological melioidosis	7	3%	16	1%	0.054
Osteomyelitis	6	3%	8	1%	0.005
Other **	7	3%	24	2%	N/A
Clinical Outcomes					
Bacteraemia	99	49%	657	61%	0.041
Septic shock	38	19%	243	23%	0.225
Required intensive care	50	25%	270	25%	0.895
Ventilated	27	13%	171	16%	0.352
Died initial illness	16	8%	122	11%	0.010

* For this study, review of specific potential exposure events in the wet season was not conducted. ** Other presentations described in case reports rare and atypical forms of melioidosis, such as mycotic aneurism, parotitis, pericarditis and abdominal masses.

## Data Availability

Individual patient data are not available, but a data dictionary can be provided upon request.

## Supplementary Materials

The following supporting information can be downloaded at: https://www.mdpi.com/article/10.3390/pathogens15070726/s1. Table S1. Parameter estimates for GAM models predicting (A) the probability of a dry season case-day across the dry season (binomial GAM) and (B) annual dry season melioidosis incidence rates over time (negative binomial GAM). Effective degrees of freedom (edf), chi-square statistics and *p* values are reported for each smooth term.

## Abbreviations

The following abbreviations are used in this manuscript:

*B. pseudomallei*	*Burkholderia pseudomallei*
DPMS	Darwin Prospective Melioidosis Study
BOM	Australian Bureau of Meteorology
NT	Northern Territory
ABS	Australian Bureau of Statistics
