# Supplementary material for: Dry Season Melioidosis in the Tropical North of Australia
Source: Pathogens. 2026 Jul 9;15(7):726. doi: 10.3390/pathogens15070726 (PMC13414569; doi:10.3390/pathogens15070726)
Supplement: Supplementary file 1 [file pathogens-15-00726-s001.zip › Supplementary Table S1.pdf]

Table S1. Parameter estimates for GAM models predicting (A) the probability of a dry season case-day across the dry season (binomial GAM) and (B) annual dry season melioidosis incidence rates over time (negative binomial GAM). Effective degrees of freedom (edf), chi-square statistics and P values are reported for each smooth term.

**Table S1A**						
	Estimate	SE	t	edf	Chi-square	p-value
**(Intercept)**	-4.3914	0.1361	-32.27			<0.001
**s(rain_mm_prev_long)**				1.00	1.92	0.166
**s(day_in_dryseason)**				2.43	15.5	0.002
**s(Year)**				2.33	23.40	<0.001

**Table S1B**						
	Estimate	SE	t	edf	Chi-square	p-value
**(Intercept)**	-10.9	0.14	-77.85			<0.001
**s(wet_rain_total)**				1.00	0.37	0.544
**s(Year)**				1.98	9.82	0.011

Note: *SE*, standard error; *t*, T statistics; *edf*, estimated degrees of freedom; *s()*, smooth terms
